# **Next**-**generation sequencing of mitochondrial targeted AAV transfer of human *ND4* in mice**

**Published:** 2013-07-14

**Authors:** Hong Yu, Arpit Mehta, Gaofeng Wang, William W. Hauswirth, Vince Chiodo, Sanford L. Boye, John Guy

**Affiliations:** 1 Bascom Palmer Eye Institute, University of Miami, Miami, FL; 2 Hussman Institute for Human Genomics, University of Miami, Miami, FL; 3 Department of Ophthalmology, University of Florida, Gainesville, FL

## Abstract

**Purpose:**

To determine the effects of mitochondrial targeting sequence (MTS) modified AAV gene delivery of wild-type human NADH dehydrogenase subunit 4 (*ND4*), mutated in most cases of the blinding disease Leber hereditary optic neuropathy (LHON), on the host mouse mitochondrial genome.

**Methods:**

We injected a modified self-complementary (sc) AAV vector, to which we appended the cytochrome oxidase subunit 8 (COX8) leader to one of the three capsid proteins (VP2) comprising the protein shell of the AAV virion, into the mouse vitreous to deliver the human *ND4* gene under the control of a mitochondrial heavy strand promoter (HSP) directly to the mitochondria of the mouse retina. Control viruses consisting of scAAV lacking the COX8 targeting sequence and containing human *ND4*, or scAAV containing *GFP*, were also vitreally injected. Using next-generation sequencing of mitochondrial DNA extracted from the pooled mouse retinas of experimental and control eyes, we tested for the presence of the transferred human *ND4*, and any potential recombination of the transferred human *ND4* gene with the endogenous host mitochondrial genome.

**Results:**

We found hundreds of human *ND4* DNA reads in mitochondrial samples of MTS AAV-*ND4*-injected eyes, a few human *ND4* reads with AAV-*ND4* lacking the MTS, and none with AAV-*GFP* injection. Putative chimeric read pairs at the 5′ or 3′ ends of human *ND4* showed only vector sequences without the flanking mouse sequences expected with homologous recombination of human *ND4* with the murine *ND4*. Examination of mouse mitochondrial *ND4* sequences for evidence of intra-molecular small-scale homologous recombination events yielded no significant stretches greater than three to four nucleotides attributable to human *ND4*. Furthermore, in no instance did human *ND4* insert into other non-homologous sites of the 16 kb host mtDNA.

**Conclusions:**

Our findings suggest that human *ND4* remains episomal in host mitochondria and is not disruptive to any of the endogenous mitochondrial genes of the host genome. Therefore, mitochondrial gene transfer with an MTS-AAV is non-mutagenic and likely to be safe if used to treat LHON patients with mutated *ND4*.

## Introduction

The clinical features of Leber hereditary optic neuropathy (LHON), a degenerative vision disorder, were first described in 1871 [1]. LHON usually presents as a bilateral loss of central vision that typically progresses over weeks to months without pain, until vision deteriorates to around 20/400 in both eyes. The mean age of onset is in the mid-20s, although the range is extremely broad. Initially, the optic disc may become hyperemic and elevated, but in many cases it remains normal in appearance until the development of optic disc pallor. More than 100 years later (1988), LHON became the first disease for which a point mutation in mitochondrial DNA (mtDNA) was identified [2]. It is now well known that human mtDNA encodes 37 genes that are essential for cell viability, in total (2 for rRNAs, 22 for tRNAs and 13 for proteins). These genes are buffered against the effect of mutations because a somatic, or non-reproductive, cell typically contains around 1,000 copies of mtDNA. Although most cells are apparently uniform with respect to mtDNA composition, mammalian cells can tolerate a significant fraction of aberrant mtDNA, a condition called heteroplasmy, and retain respiratory energy function. LHON is typically homoplasmic with 100% mutant mtDNA in leukocytes obtained from the peripheral blood.

Most LHON cases are associated with mutations in one of three mitochondrial genes for subunits of NADH ubiquinone oxidoreductase, which is complex I of the mitochondrial respiratory chain [3,4]. This enzyme contains seven subunits encoded by mtDNA that are intimately associated with the inner mitochondrial membrane and 35 subunits that are encoded by nuclear DNA and imported into the organelle [5]. Approximately 50% of LHON patients have the G11778A mutation; the rest have the G3460A mutation, which affects the *ND1* gene, or the T14484C mutation, which affects the *ND6* gene. These three mutations are considered the primary causes of LHON, and each presents a significant risk of permanent visual loss. All are associated with focal degeneration of retinal ganglion cells (RGCs). Two of the primary LHON mutations, G3460A and T14484C, lead to an 80% reduction in NADH dehydrogenase activity in mitochondria isolated from platelets of patients [6,7]. However, mitochondria isolated from G11778A cells show near-normal activity of complex I and most other components of the respiratory chain [8]. We discovered that ND4 mutant cells have severe reductions in ATP synthesis, even though mild reductions in complex I activity appeared insufficient to induce disease [9]. With the recent failure of idebenone (a Coenzyme Q analog with antioxidant properties) to show significant efficacy in the primary outcome measure to improve vision in a masked randomized clinical trial [10], there remains no effective treatment for LHON or, for that matter, any of the myriad diseases associated with mutated mtDNA [11-13].

Therapies for LHON in common with all disorders caused by mutated mtDNA are inadequate, in large part because of the barrier in delivering DNA into the organelle. We have broken through this barrier [14]. Our pioneering studies showing that DNA can be efficiently introduced into mitochondria in vitro and in vivo to correct a biochemical defect in respiration and preserve visual function caused by a mitochondrial DNA mutation opened the door for directed gene therapy to the organelle. Specifically, we redirected the AAV virion to mitochondria rather than to its typical target, the nucleus, by addition of a mitochondrial-targeting sequence (MTS) to the capsid (the protein shell of the virus). Transmission electron micrographs revealed that the MTS virion composed of VP1, VP2, and VP3 capsid proteins uncoats adjacent to the outer mitochondrial membrane where the linear self-complementary human *ND4* DNA and the degraded capsid protein with GFP attached to the MTS enter the organelle through the import channels [15], resulting in expression of the ND4FLAG protein detected by anti-FLAG immunogold that incorporates into respiratory complexes, as shown by blue native PAGE [14]. This delivery of the wild-type human *ND4* gene in the mitochondrial genetic code into the organelle rescued the defective energy production of G11778A LHON hybrid cells.

We then applied our MTS AAV technology to animals by testing for the presence and expression of the human *ND4* gene in the adult rodent visual system that prevented visual loss and atrophy of the optic nerve induced by coadministration of a second AAV containing the allotopic human mutant ND4 allele; i.e., the arginine to histidine transition at amino acid 340 (R340H). Allotopic R340H ND4 AAV injected into the vitreous of rodents was previously demonstrated by us to cause optic nerve swelling, followed later by atrophy of the nerve along with loss of RGCs [16]. The same result was also reported later by another independent group [17]. In prior work with the MTS AAV, we also constructed a mutant *ND4* gene that, when exchanged for the wild-type *ND4* in the scAAV construct and subsequently injected into the mouse vitreous, induced visual loss and optic atrophy [18]. These are the hallmarks of human LHON [19].

In our prior work with the MTS AAV [14], PCR of mitochondrial DNA extracted from the retinas and optic nerves of MTS AAV-*ND4*-injected eyes suggested possible homologous recombination of human *ND4* with the host mouse *ND*4 mtDNA. Here we used next-generation sequencing to test for the safety of MTS-AAV gene transfer by looking for any adverse recombination of the MTS AAV-transferred human *ND4* with the host mitochondrial genome of the mouse.

## Methods

### Plasmids and AAVs

Human wild-type *ND4* was fused in frame with FLAG and cloned into a self-complementary (sc) AAV backbone under the control of the mitochondrial heavy strand promoter (HSP) to give sc-HSP*-ND4FLAG*. VP2COX8GFP was generated by first linking the 23 amino acid MTS presequence of cytochrome oxidase subunit 8 (COX 8) to *GFP* and then inserting the whole cassette into the VP2 capsid of AAV at the Eag I site at residue 138. Self-complementary AAV2/sc-HSP-*ND4FLAG* or a control scAAV2-*GFP* was produced by the plasmid cotransfection method. The plasmids were amplified and purified by cesium chloride gradient centrifugation and then packaged with the VP2COX8GFP or standard VP2 plus VP1, VP3, and the helper plasmid PXX6 (threefold excess) into AAV2 recombinant virus by transfection into 293 cells as described previously [14]. In brief, the crude iodixanol fractions were purified using the Pharmacia AKTA FPLC system (AKTA; Amersham Pharmacia, Piscataway, NJ). The virus was then eluted from the column with 215 mM NaCl, pH 8.0, and the rAAV peak was collected. The rAAV-containing fractions were then concentrated and buffer exchanged in Alcon BSS with 0.014% Tween-20, with a Biomax 100 K concentrator (Millipore, Billerica, MA). The virus was then titered for DNase-resistant viral genomes by real-time PCR relative to a standard. Finally, the purity of the virus was validated by silver-stained sodium dodecyl sulfate–PAGE, assayed for sterility and lack of endotoxin, and then aliquoted and stored at −80 °C.

### Animals

All animal procedures were performed in accordance with the National Institutes of Health Guide for Care and Use of Laboratory Animals and the ARVO Statement for the use of Animals in Ophthalmic and Vision Research and were approved by the University of Miami IACUC. For the intraocular injection of recombinant AAV, three month old DBA/1J mice were sedated by inhalation with 1.5% to 2% isofluorane. A local anesthetic (proparacaine HCl) was applied topically to the cornea, and then a 32-gauge needle attached to a Hamilton syringe was inserted through the pars plana. One microliter of scAAV2/COX8VP2*ND4FLAG* (1.01 x10^11^ VG/ml; n=3 mice), scAAV2/VP2*ND4FLAG*, (n=3 mice), or scAAV-*GFP* (1.03 X 10^12^ VG/ml; n=3 mice) was injected into the vitreous body of both eyes. Animals were euthanized nine days post-injection (CO_2_ inhalation followed by IM injection of euthanisol solution). Mitochondrial-enriched fractions were isolated from retinal tissues and used to extract DNA using the DNeasy blood and tissue kit (Qiagen,Valencia, CA) according to the manufacturer’s instructions as previously described [14]. Retinal mitochondrial DNA samples from six eyes were pooled for each construct and submitted for next-generation sequencing.

### NGS and Bioinformatic Analysis

Library construction was performed according to the Illumina ® TruSeq™ DNA Sample Preparation Guide (Hayward, CA). The final median insert size was 250–300 bp. Samples were bar-coded to allow for multiplexing. Cluster generation took place on the Illumina cBot according to the manufacturer’s recommendations. Sequencing occurred on the Illumina HiSeq2000 using the reagents provided in the Illumina TruSeq PE Cluster Kit v3 and the TruSeq SBS Kit – HS (200 cycle) kit. Sequence alignment was performed with the Burroughs-Wheeler Aligner (BWA; Cambridge, UK) with default parameter settings. Two 100 bp paired-end reads from the Illumina Hiseq2000 were separately aligned to the human (hg19) and mouse (mm9) genomes, as well as the *ND4* AAV constructs. Human and mouse mitochondrial alignments were subsequently extracted from their respective BAM (tab-delimited text file that contains sequence alignment data) files. To identify potential mitochondrial insertions of the construct, we first searched for evidence of paired-end reads, wherein one of the paired ends mapped to the construct sequence (human *ND4* or elsewhere in construct) and the other pair was aligned within the mitochondrial genome of the mouse. Using BWA mapping results from both the human and mouse alignments, along with custom PERL scripting, we identified all putative instances of chimeric mapping events for additional manual evaluation of possible microscale homologous recombination events between the transfected human construct and mouse mitochondria. For these analyses, it was only possible to assess microscale recombination events at regions of homology between the construct and the mouse mitochondria, such as the *ND4* gene. As described above, BWA alignment was first performed targeting the mouse mm9 genome. All mitochondrial alignments were subsequently extracted from the resulting BAM files. At each homologous position where the human and mouse bases differed (IE, at informative nucleotide positions), we examined the Illumina sequence data for instances where the mouse mitochondrial sequence matched the human. Since, by chance alone, any sequencing error or mutation on the mouse mitochondria had a 1/3 chance of matching the corresponding human base, only stretches of multiple human bases were taken as evidence for putative homologous recombination events and selected for further manual examination.

## Results

### Detection of human *ND4*

**Figure 1:**
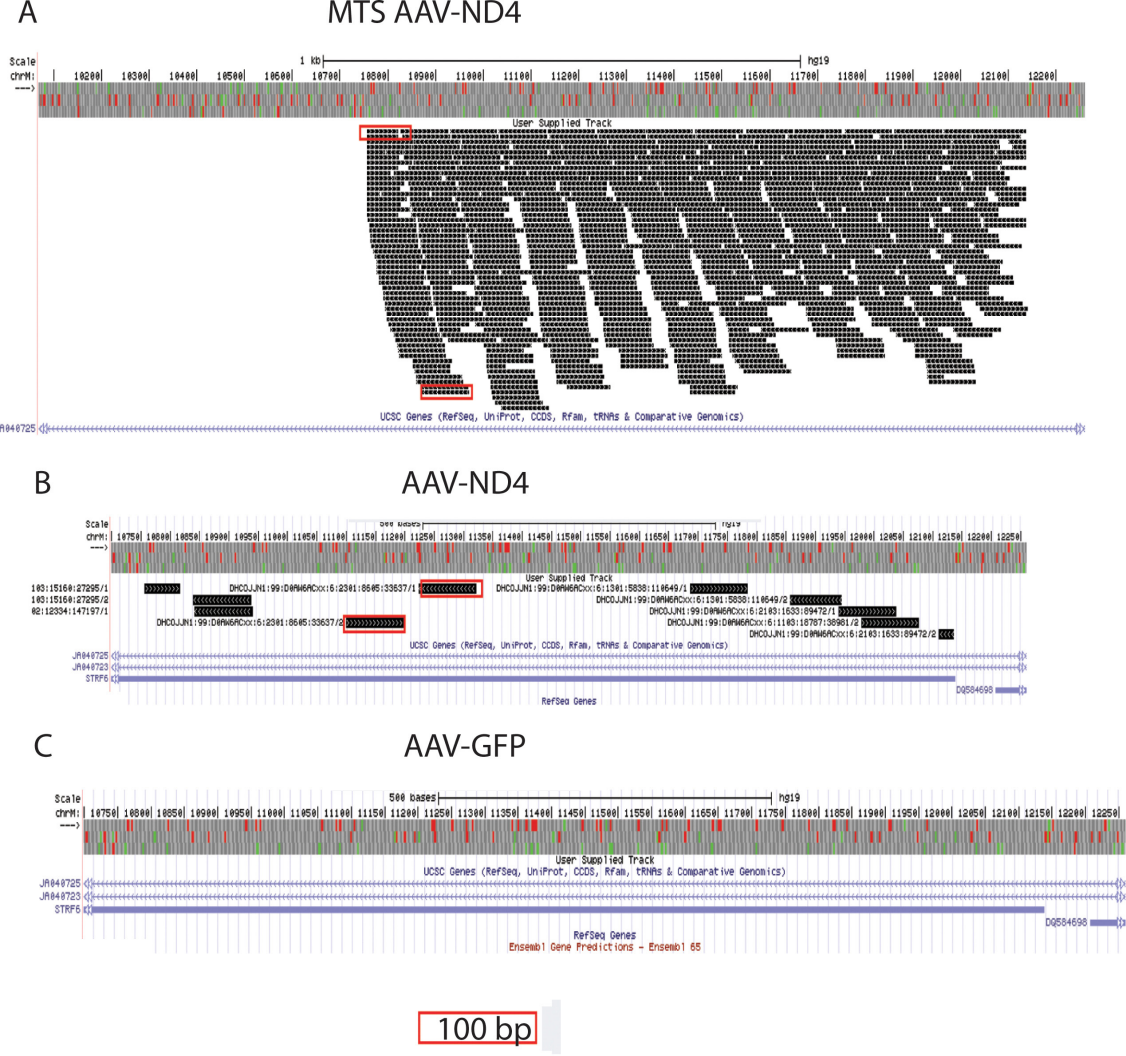
Human *ND4* Sequencing. **A**: Over 500 reads 100 bases long spanned the entire approximately 1.4 kb of human *ND4* in retinal samples injected with the MTS AAV containing human *ND4* (MTS AAV-*ND4*). **B**: Only ten reads of human *ND4* were identified in the retinal samples injected with the untargeted AAV containing human *ND4* (AAV-*ND4)*. **C**: No human *ND4* reads were detected in samples of mouse eyes injected with AAV-*GFP*. The red boxes illustrate100 bp read length.

If the MTS-AAV delivered human *ND4* to mitochondria, then we should be able to detect human *ND4* DNA in infected mouse retinas. Indeed, we found 579 sequencing reads (100 bp long) spanning the entire 1377 nucleotides of human *ND4* in the mouse retinal samples injected with the MTS AAV containing human *ND4* (scAAV2/COX8VP2; Figure 1A). In contrast, ten reads of human *ND4* were identified in the retinal samples injected with the untargeted AAV containing human *ND4* (scAAV2/VP2), which typically delivers its DNA to the nucleus (Figure 1B). Lastly, no human *ND4* reads were detected in samples of mouse eyes injected with scAAV-*GFP* (Figure 1C). Thus, the MTS AAV efficiently delivered human *ND4* to mouse mitochondria and the untargeted AAV did not.

### Lack of chimeric end pair reads

**Figure 2:**
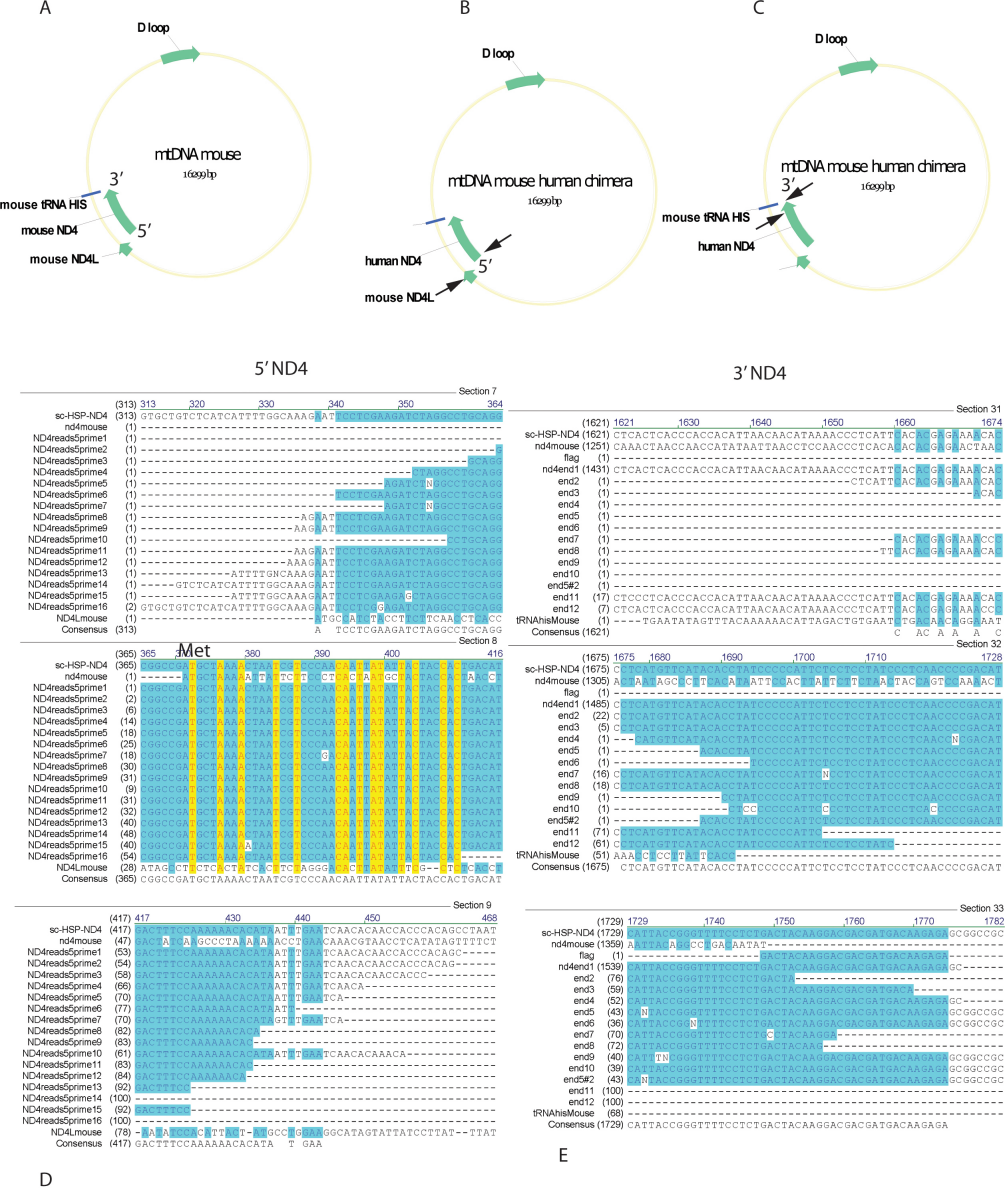
Lack of homologous recombination. **A**: An illustration of the mouse mitochondrial DNA shows that murine *ND4* is flanked by two genes. **B**: An illustration of the mouse mitochondrial DNA shows that the adjacent gene encoding for *ND4L* (arrow) is at the 5′ end of murine *ND4* (arrow). **C**: An illustration of the mouse mitochondrial genome shows that the gene for the tRNA histidine (arrow) is at the 3′ end of mouse *ND4* (arrow). **D**: Alignment of 5′ human *ND4* reads for mouse human chimeras shows only AAV vector sequences upstream of the start methionine codon (ATG) for *ND4*. If there were homologous recombination of human *ND4* into the mouse mitochondrial genome replacing murine *ND4*, then nucleotides for mouse *ND4L* would be expected to be adjacent to the ATG methionine codon. **E**: Alignment of the 3′ human *ND4* reads shows the FLAG epitope that we appended to human *ND4* for immunodetection and adjacent vector sequences, but not the tRNA histidine that would have indicated homologous recombination. Sc-HSP-ND4=self-complementary AAV plasmid containing the heavy strand promoter (HSP) driving human *ND4.*

Next, we tested whether human *ND4* had replaced the host *ND4*. Murine *ND4* is flanked by two genes, *ND4L* and tRNA histidine (tRNA His; Figure 2A). The adjacent gene encoding *ND4L* is at the 5′ end of murine *ND4* (Figure 2B). The gene for the tRNA His is at the 3′ end of mouse *ND4* (Figure 2C). If there were homologous recombination of human *ND4* into the mouse mitochondrial genome, then we would expect to find sequences of mouse *ND4L* at the 5′ end of human *ND4* and/or sequences of tRNA histidine at the 3′ end of human *ND4*. Examination of all putative chimeric read pairs at the 5′ end of human *ND4* revealed only vector sequences, not mouse *ND4L (*Figure 2D*)*. Thus, there was no evidence for homologous recombination at the 5′ end of human *ND4*. Similarly, at the 3′ end of human *ND4*, we found the FLAG epitope that we had appended to human *ND4* for immunodetection. Adjacent vector sequences were also detected (Figure 2E), but not the tRNA histidine that is immediately downstream of mouse *ND4.* Thus, human *ND4* did not replace mouse *ND4* in the host mitochondrial genome.

### Lack of small-scale homologous recombination events

Next, we looked for subtle evidence of small-scale recombination events of human *ND4* insertion within the mouse *ND4*. Examination of mouse mitochondrial *ND4* sequences for evidence of intra-molecular small-scale homologous recombination events yielded no significant stretches greater than four nucleotides attributable to human *ND4* integration. At the sites where human and mouse nucleotides differ, there were sporadic instances where single bases matched the human sequence in 156 instances for MTS AAV-*ND4*, 127 instances for untargeted AAV-*ND4*, and 91 instances for AAV-*GFP*. In addition, there were 13 instances where two adjacent bases matched the human *ND4* in the MTS AAV-*ND4* samples. There were nine instances where two adjacent bases matched the human *ND4* in the untargeted AAV-*ND4* samples. There were five instances where two adjacent bases matched human *ND4* in the AAV-*GFP* injected samples.

**Figure 3:**
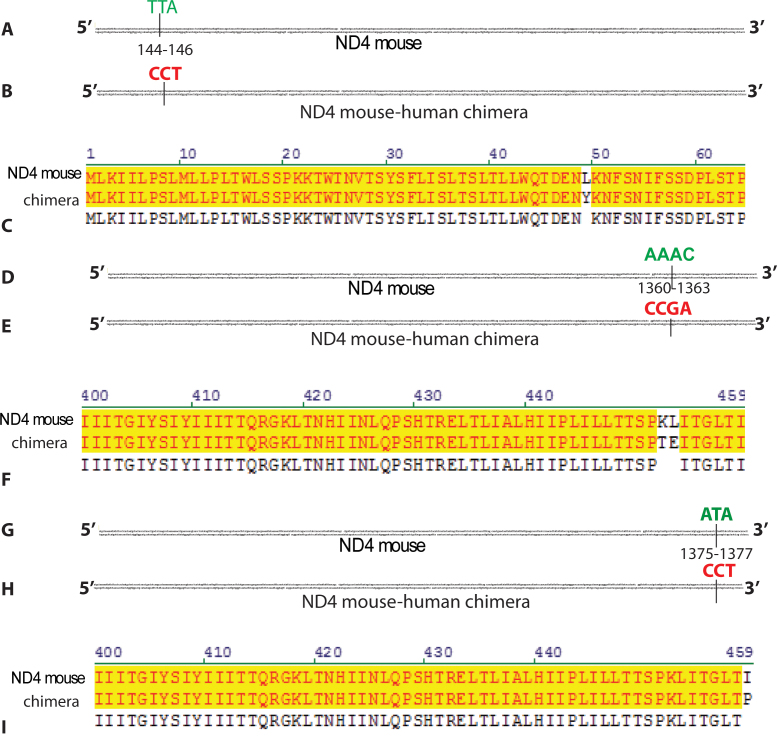
Small-scale recombination. **A**: An illustration shows the nucleotides TTA at positions 144–146 of mouse *ND4*. **B**: An illustration shows the CCT codon of human *ND4* occupying positions 144–146 of murine *ND4* in next-generation sequencing reads of mouse *ND4* from eyes injected with MTS AAV-*ND4*. **C**: An alignment of *ND4* protein amino acids shows that the CCT substitution for TTA results in a transition of tyrosine (Y) for leucine (L) at amino acid 49 of mouse *ND4*. **D**: An illustration shows nucleotides AAAC at positions 1360–1363 of mouse *ND4*. **E**: An illustration shows human nucleotides CCGA at positions 1360–1363 of mouse *ND4* in MTS AAV-*ND4*-injected eyes. **F**: An alignment of *ND4* amino acids shows that the human CCGA substitution for mouse AAAC would result in a transition of threonine (T) for lysine (K) at amino acid 452, as well as a transition of glutamic acid (E) for leucine (L) at amino acid 453. **G**: An illustration shows the nucleotides ATA at positions 1375–1377 of mouse *ND4*. (H) An illustration shows the nucleotides ATA of human *ND4* at positions 1375–1377 of mouse *ND4* next-generation sequencing reads in MTS AAV-*ND4*-injected eyes. **I**: Substitution of human CCT for murine ATA would result in a transition of proline (P) for isoleucine (I) at the last amino acid 459 of mouse *ND4*.

However, in samples derived from MTS AAV-injected eyes, we found two instances where three bases in a row of the mouse *ND4* matched the human sequence and a single instance where four bases in a row matched human *ND4*. The first was at positions 144–146 of mouse *ND4*, where nucleotides TTA (Figure 3A) were replaced by nucleotides CCT of human *ND4* with MTS AAV-*ND4* injection (Figure 3B). Such a recombination event would result in a transition of tyrosine for leucine at amino acid 49 of mouse *ND4* (Figure 3C). Next, at positions 1360–1363 of mouse *ND4*, nucleotides AAAC (Figure 3D) were replaced by nucleotides CCGA of human *ND4* with an MTS AAV-*ND4* injection (Figure 3E). Such a recombination event would substitute threonine for lysine at amino acid 452 and glutamic acid for leucine at amino acid 453 in the murine *ND4* (Figure 3F). Lastly, at positions 1375–1377 of mouse *ND4* nucleotides, ATA (Figure 3G) were replaced by nucleotides CCT of human *ND4* in retinal samples from AAV-*ND4*-injected eyes (Figure 3H). Such a recombination event would result in a transition of proline for isoleucine at amino acid 459 of mouse *ND4* (Figure 3I). However, these three- and four- nucleotide recombination events were seen in one or two mouse *ND4* reads out of a total of 4000 reads. This is well below the anticipated 0.1% sequencing error rate expected from NGS, and most likely an artifact of the method. Still, these small-scale recombinations were only detected in the MTS AAV-*ND4*-injected eyes.

### Lack of insertions of human ND4 into non-homologous sites of mouse mtDNA

**Figure 4:**
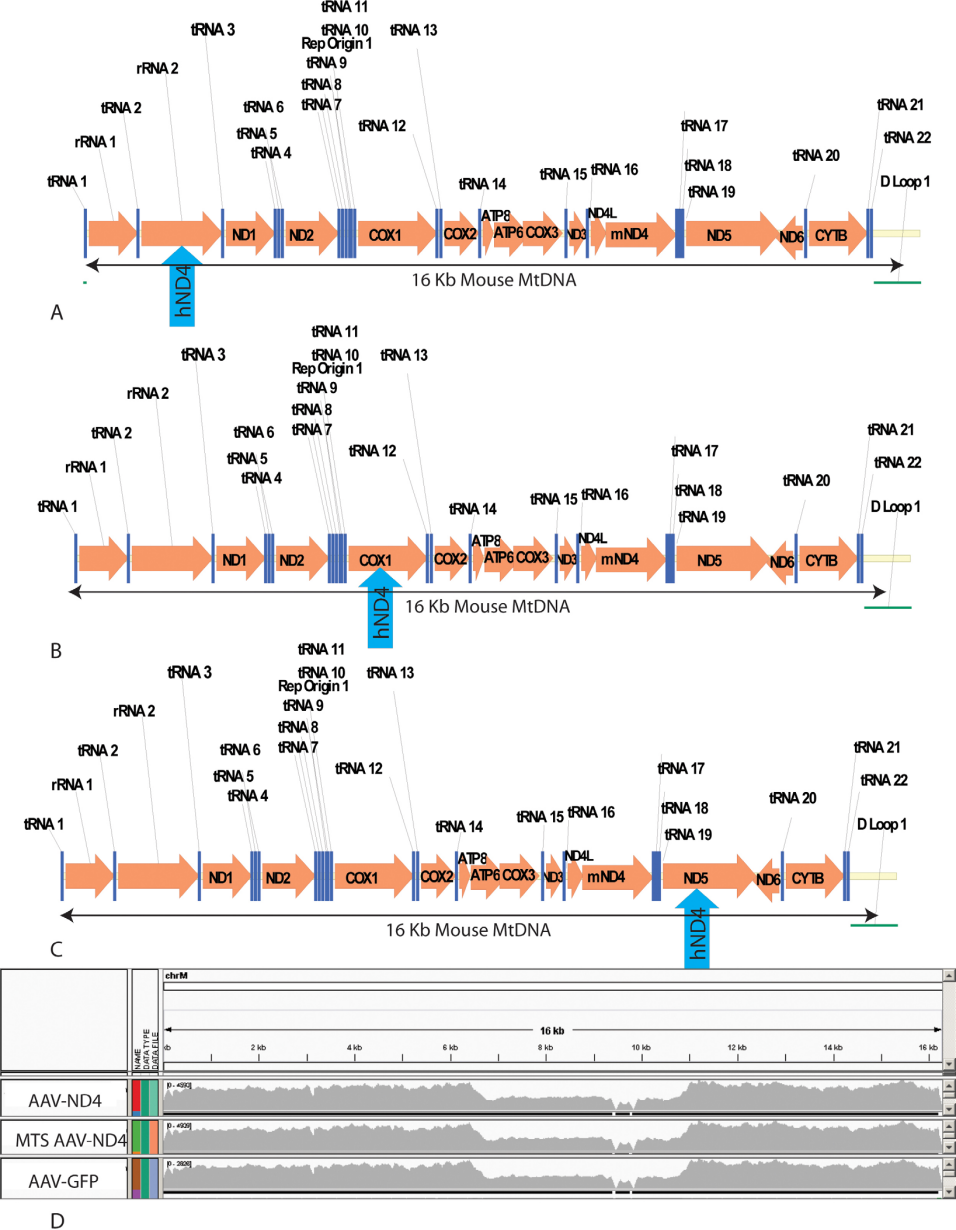
Lack of non-homologous recombination. **A**: An illustration of the 16 kb mouse mitochondrial genome shows potential insertion of human *ND4* (blue arrow) between the tRNAs and rRNAs, cytochrome oxidase subunit 1 (**B**), or *ND5* (**C**). **D**: Next-generation sequencing reads of the entire mouse mitochondrial genome of AAV-*ND4-*, MTS-AAV-*ND4-*, or AAV-*GFP*-injected eyes were identical and showed no insertions of human *ND4* bases into non-homologous sites of the host genome.

Next, we tested whether human *ND4* integrated into non-homologous sites in the mouse mitochondrial genome; because the insertion could potentially disrupt mitochondrial encoded tRNAs, rRNAs or proteins such as cytochrome oxidase subunit 1 (Figure 4B), ND3 or ND5 (Figure 4C). Here we found 43,079 one hundred bp mouse mitochondrial DNA reads with MTS-AAV-*ND4* injection, 45,505 reads with AAV-*ND4* lacking the MTS, and 26,672 reads with AAV-*GFP*. Spanning the 16,000 bases of the mouse mitochondrial genome, in no instance did human *ND4* insert into any site of mouse mtDNA (Figure 4D). Thus, the presence of transferred human ND4 did not disrupt the host mitochondrial genome.

### Lack of mitochondrial depletion

As our findings indicated human *ND4* remained episomal, we looked for any evidence of mitochondrial depletion potentially induced by preferential replication of the smaller human *ND4* (1.4 kb). Looking at the ratio of the entire mitochondrial genome reads to the nuclear genome reads of the mouse, ratios were 0.00963 for the MTS AAV-*ND4* relative to 0.01274 for the untargeted AAV-*ND4*; However, the ratio for the AAV-*GFP* injected eyes was 0.005665. Relative to the AAV-*GFP* control, mitochondrial/nuclear read ratios increased 170% for MTS AAV-*ND4* and 225% for untargeted AAV-*ND4*. Taken together, we found no evidence for mitochondrial DNA depletion at 9 days induced by the presence of episomal human AAV-*ND4* in retinal mitochondria.

## Discussion

Our results here suggest that the MTS AAV-transferred human *ND4* remains episomal in mitochondria of the mouse retina. Despite the mixing of human and mouse mitochondrial genes in RGCs, we found no evidence for homologous recombination of transferred human *ND4* with the host *ND4* using highly sensitive next-generation sequencing technology. Our previous PCR data had shown sequences of the histidine tRNA at the 3′ end of human *ND4* that suggested homologous recombination [14]. However, looking closely at the 3′ break point of transferred human *ND4,* we found only the adjacent FLAG epitope and vector sequences and not the histidine tRNA that is adjacent to the 3′ end of murine *ND4* in the host mitochondrial genome. Similarly, at the other end adjacent to the start methionine of human *ND4*, we found only vector sequences and not the *ND4L* gene adjacent to the start methionine of mouse *ND4*. Therefore, human ND4 did not replace mouse ND4 of the host mitochondrial genome. Replication of the human ND4 DNA delivered by our MTS-AAV may have occurred by the GC rich region of the inverted terminal repeat (iTR) of the AAV vector that is highly homologous to the GC rich control sequence box 2 (CSB2) in the origin of replication region of the host mitochondrial genome.

As exchanges of mitochondrial DNA between different plant species, some animals, and (very rarely) in humans have been reported [20-26], we looked closely for small-scale recombination events consisting of only a few nucleotides in a row. Here we did find several instances of small-scale recombination events, where three or four nucleotides in a row of human *ND4* were sequenced in the mouse *ND4* samples obtained from MTS AAV-*ND4*-injected eyes, but not with standard untargeted AAV-*ND4* or AAV-*GFP*. Such events, if frequent, could potentially result in mutated host ND4 proteins. Fortunately, this is unlikely, as these small-scale recombinations were seen in only a few reads out of several thousand. Still, AAV serotype 2 (to which we attached the MTS and injected into the vitreous cavity) is directed primarily to RGCs, the cell type affected in LHON [14]. It should also be pointed out here that mitochondrial DNA was extracted from the entire retina, where RGCs comprise less than 5% of all retinal cells [27]. Therefore, the relatively smaller targeted cell population, RGCs, likely contributed to the more than 100-fold lower human *ND4* reads relative to total murine *ND4* reads. In that context, small-scale homologous recombinations could exert a deleterious effect as demonstrated by Fan et al. [28], who found mtDNA homologous recombination in mouse cell lines that had induced pathogenic missense and frame shift mutations. It is for this reason that we looked closely at the potential effects of our three small-scale insertions of human *ND4* nucleotides into the mouse *ND4*, finding only non-synonymous amino acid changes that are of no known pathogenicity.

Finally, sequencing of the entire mouse mitochondrial genome failed to reveal insertions of the human *ND4* into non-homologous sites that could have disrupted mitochondrial protein synthesis and oxidative phosphorylation. In our study, we used 3 month old mice that showed no evidence of mitochondrial deletions characteristically seen in cells and tissues of older mammals including humans [29-31]. Such breaks in the mitochondrial genome associated with aging could potentially result in insertions of MTS-transferred human *ND4* into the mouse mitochondrial genome at non-homologous sites [32]. Taken together, our findings indicate that MTS AAV transfer of human DNA is unlikely to be mutagenic in the mouse eye and thus unlikely to contribute to the optic neuropathy induced by MTS AAV transfer of mutant ND4 in this model [18]. Moreover, the heteroplasmy induced by the mixing of two different mammalian mitochondrial genomes does not result in recombination, at least during the short term (nine days after intravitreal injection). Our study shows that MTS AAV transfer of wild-type human *ND4* is nonpathogenic to host mitochondria, and supports our previous studies that indicate an MTS AAV-*ND4* can be used to rescue visual loss and optic neuropathy induced by the mutant *ND4* allele responsible for most cases of LHON [14]. It remains to be determined by clinical trials whether our MTS AAV-ND4 will benefit LHON patients with a) chronic or b) acute visual loss or c) fellow eyes with good vision that are destined to go blind in approximately two months after visual loss in the opposite eye and d) how long the beneficial effect will last. If replication of the linear human ND4 DNA delivered to the mitochondria by the MTS AAV shown in our previous work [14], occurs by a mechanism similar to that of 7S mitochondrial DNA [33] or perhaps by the replication element of the AAV iTR [34], then a single treatment may be sufficient for sustained rescue of visual function.
